# An ethnographic study of the effects of cognitive symptoms in patients with major depressive disorder: the IMPACT study

**DOI:** 10.1186/s12888-017-1523-8

**Published:** 2017-11-21

**Authors:** Bjarke Ebert, Kamilla Miskowiak, Morten Kloster, Jon Johansen, Cara Eckholm, Torbjörn Wærner, Mads Holme, Louise Meldgaard Bruun

**Affiliations:** 1 H. Lundbeck A/S, Ottiliavej 9, 2500 Valby, Denmark; 2 Psychiatric Centre Copenhagen, Copenhagen University Hospital, Rigshospitalet, Blegdamsvej 9, 2100 København Ø, Denmark; 3 ReD Associates, Kronprinsessegade 20, DK-1306 København, Denmark; 4 Aarhus University Hospital, Skovagervej 2, 8240 Risskov, Denmark

**Keywords:** Major depressive disorder, Cognitive symptoms, Qualitative study, Brazil, Canada, China, France, Germany

## Abstract

**Background:**

The manifestation of major depressive disorder (MDD) may include cognitive symptoms that can precede the onset of MDD and persist beyond the resolution of acute depressive episodes. However, little is known about how cognitive symptoms are experienced by MDD patients and the people around them.

**Methods:**

In this international (Brazil, Canada, China, France, and Germany) ethnographic study, we conducted semi-structured interviews and observations of remitted as well as symptomatic MDD patients (all patients self-reported being diagnosed by an HCP and self-reported being on an antidepressant) aged 18–60 years with self-reported cognitive symptoms (*N* = 34). In addition, participating depressed patients’ close family or friends (*N* = 31) were interviewed. Separately recruited from depressed participants, work colleagues (*N* = 21) and healthcare providers (HCPs; *N* = 13) of depressed individuals were interviewed.

**Results:**

Key insights were that: (1) patients were generally unaware that their cognitive symptoms were linked to their depression and, instead, attributed these symptoms to negative aspects of their person (e.g., age, separate disease, laziness, exhaustion); (2) cognitive symptoms in MDD appeared to negatively impact patients’ social relationships and patients’ ability to handle daily tasks at work and at home; (3) patients’ cognitive symptoms also impacted relationships with family members and coworkers; (4) patients’ cognitive symptoms increased stress and feelings of failure, which in turn seemed to worsen the cognitive symptoms, thereby creating a destructive cycle; and (5) although HCPs recommended that patients re-engage in everyday activities to help overcome their depression, cognitive symptoms seemed to impede such functional recovery.

**Conclusions:**

Taken together, these findings highlight a negative impact of patients’ cognitive symptoms on their social functioning, work performance, and quality of life on the people close to them, and consequently on the degree of functional recovery after depression.

**Electronic supplementary material:**

The online version of this article (10.1186/s12888-017-1523-8) contains supplementary material, which is available to authorized users.

## Background

### A case study

Clara, a 23-year-old student, had just begun a master’s degree at a French university when she suddenly fell into what she describes as a “third dimension.” Her mood lowered, she was stressed and irritable, and she started getting into fights with her mother. Her brain seemed to slow down and everyday tasks, such as homework, became insurmountable. “I felt like I was stoned or drunk, or something,” says Clara, reflecting on her mental state throughout her first months at university. Clara fretted but took no action—until one day when she woke up with her head spinning. The vertigo was so severe that she could not get out of bed, prompting her to make a rush appointment with her general practitioner. After a couple of sessions, Clara was diagnosed with major depressive disorder (MDD) and prescribed medication.

For the following 2 months, Clara did not attend classes; she quit her part time job as a secretary at a bank. “I was exhausted physically and intellectually, at every level,” she stated. She was bedridden with a terrible mood and with little mental capacity or willpower. By the time of our visit 6 months later, Clara was back at university and at work, but her return to normality had been riddled with frustration. Her mood stabilized, but her cognitive abilities were still plagued by abnormal lapses. Her memory was shoddy: “It still happens that I ask a question, the person answers, and I ask it again 2 minutes later,” and her ability to concentrate was in a state of disrepair: “I was in a 4-hour exam, and for 3 of the hours I didn’t write anything at all,” she lamented.

How do we make sense of Clara’s struggle to recover from depression? Historically, MDD has been typified by its affective symptoms, related to lowered mood. However, recent studies have shown that a range of cognitive symptoms is often present during MDD and can persist after symptomatic remission from depression [1, 2].

### Burden of depression and cognitive symptoms

MDD is a common psychiatric disorder, with an estimated point prevalence of 4.7% of the global population [3]. The negative consequences are profound and impact health-related quality of life, workplace performance and sick days (presenteeism and absenteeism, respectively), and interpersonal relationships [4–9]. The global economic toll of MDD was estimated to be US$800 billion in 2010 and is rising [10]. In the US alone, the incremental economic burden of individuals with MDD in 2010 was estimated to be US$210.5 billion, with approximately half of the total incremental costs associated with the workplace [11].

Cognitive symptoms are a common feature of MDD that contributes to this large economic burden. In particular, cognitive complaints in MDD can be broad and non-specific, with deficits observed across processing speed, attention, learning and memory, and executive functioning [1, 2]. Evidence suggests that cognitive symptoms directly influence workplace performance (perhaps to an even greater extent than affective symptoms) and directly contribute to socio-occupational disability independently of mood symptoms [12, 13]. Subtle cognitive symptoms may also precede the onset of the formal major depressive episode (MDE) and increase the risk of illness onset [1, 14]. Furthermore, deficits in processing speed, attention, learning and memory, and executive functioning may persist beyond depressive remission as residual cognitive symptoms [1, 15, 16].

Despite evidence of the inherent harms and long-lasting effects of cognitive symptoms, MDD treatment remains primarily directed at managing mood symptoms, with limited attention given to the management of associated cognitive complaints [17, 18]. Nevertheless, only a few studies have investigated how patients with MDD experience cognitive symptoms and the consequent implications of those experiences for the people around them. This ethnographic study aims to evaluate the perceived impact of cognitive symptoms on the everyday life of MDD patients and of those with whom patients are regularly in contact. The insights from this study will help inform the development of educational material for people with depression and their families, friends, and work colleagues and help lead to the development of new tools to help healthcare providers (HCPs) better identify and treat people who experience cognitive symptoms in their everyday life in connection with MDD.

## Methods

### Study design

This qualitative ethnographic study—the IMPACT study (**I**nvestigating **M**DD **P**atients’ **A**ccounts of **C**ognitive Symptoms During **T**reatment)—was conducted between May and June 2014 in major cities in Brazil, Canada, China, France, and Germany. The study utilized a patient-centric research model to capture the experience of cognitive symptoms in MDD in daily life, including their impact on patients and their partners/relatives/friends, colleagues, and HCPs. The protocol and research themes were informed by an earlier pilot study conducted in Denmark from December 2013 to January 2014. In the pilot study, we conducted 36 semi-structured interviews and observed depressed patients with cognitive symptoms (*N* = 14), their family or friends (*N* = 14), and HCPs (*N* = 8). A key observation was that participants consistently stated that their cognitive symptoms were impairing their work life. Based on this observation, this study included interviews with coworkers.

The IMPACT study was conducted following the ethical standards outlined by the ICC/ESOMAR International Code on Market and Social Research [19], which sets out global standards for self-regulation for researchers and data analysts, as well as relevant national standards for participating countries [20–23]. Patients signed consent forms and trained anthropologists conducted all interviews.

### Participants and recruitment strategies

#### Patients

Patients with MDD aged 18–60 years were recruited and screened for study participation in Brazil, Canada, China, France, and Germany. To find patients, ReD Associates, a consultancy that specializes in anthropological research, worked with experienced, local recruitment agencies that specialize in identifying patients to partake in medical studies. Once the recruitment agency confirmed a patient met all criteria, ReD conducted an additional telephone screening to re-confirm each patient’s fit for the study. In this conversation, ReD researchers checked that patients had received a relevant diagnosis by a qualified HCP and that patients were taking relevant medication for MDD (excluding those known to cause/affect cognitive symptoms). During the daylong in-home interview, the investigator confirmed the presence of the antidepressant identified during screening. At least one partner, relative, or close friend of each MDD patient accompanied the patient for a portion of the in-home interview (≥1 h). Criteria for patient participation included self-reported (1) history of an MDE within the previous year, with moderate or severe symptoms and without a diagnosis of any mental illness other than MDD; (2) memory and concentration complaints while depressed, ranging in severity from “some” to a “large” extent; and (3) to be on an antidepressant medication and be capable of naming the antidepressant. Investigators made no formal, independent diagnosis of MDD; the presence of MDD was determined by a patient’s self-reported diagnosis from an HCP, self-reported depression history, and self-reported usage of antidepressant medication. Seven patients were recruited in each country. Recruitment targets applied to each country are listed in Table 1 and were chosen to produce a diverse pool of patient experiences. Patients received a modest stipend in compensation for their time.

**Table 1:** IMPACT study entry criteria for patient participants in each country

Criteria		Targets		
Condition-related	Depression type	Mix of moderate to severe typologies		
	Memory problems	Self-reported memory problems, ranging from “some” to a “large” extent		
	Concentration problems	Self-reported concentration problems, ranging from “some” to a “large” extent		
Socio-demographic	Gender	≥3 men	≥3 women	
	Age, *years*	Minimum age, 18	≥3 patients between 30–44	Maximum age, 60
	Living situation	Mix of living: alone, with parents or siblings, with partner without children, or with partner with children		
	Education	≥4 patients with at least a college degree	≥1 patient with no more than high school education	
Work-related	Work type	Mix of workers: administrative, knowledge, people-centered, or physical		
	Sick leave	≥2 patients on sick leave	≥2 patients who have returned to work	

#### Coworkers and workplaces

Workplace participants (i.e. individuals who had worked closely with someone who had depression) were identified and recruited separately from patient participants. Recruitment targets (for each country) are listed in Table 2 and were chosen to produce a diverse pool of coworker experiences and workplace environments. Both the workplace and the employee affected by depression needed to fit the same work-type category (e.g. a knowledge worker in a company that generally does knowledge work).

**Table 2:** IMPACT study entry criteria for workplaces in each country

Criteria	Targets	
Size of workplace	≥1 company with 6–49 employees	≥1 company with ≥50 employees
Work type^a^	Mix of workplaces: administrative, knowledge, people-centered, or physical	
Relation to depressed patient	Mix of colleagues, superiors, and HR managers	
HR department	≥1 company with a dedicated HR department	≥1 company with no dedicated HR department

*HR* human resources

^a^Both the workplace and the employee affected by depression needed to fit the same category (e.g. a knowledge worker in a company that generally does knowledge work)

#### Healthcare providers

HCPs were also identified and recruited separately from patient participants. Recruitment targets (for each country) are listed in Table 3 and were chosen to produce a diverse, yet knowledgeable pool of HCP experiences and practices. All HCPs were required to be active in the management of patients with MDD and to have specialized in the field. All were required to have ≥5 years at their current practice and to diagnose and treat MDD patients on a regular basis, with ≥20% of their MDD patients identified as having cognitive symptoms. Psychiatrists and general practitioners had to prescribe antidepressants regularly and had to have prescribed a new brand or type of antidepressant ≤18 months prior to study participation.

**Table 3:** IMPACT study entry criteria for healthcare providers in each country

HCP	Clinical experience	Proportion of patients with MDD	Frequency of prescribing antidepressants	Last time prescribing new brand or type of antidepressants
Psychiatrist	≥5 years in current practice	≥20% of patients with MDD	≥4 times a week	≥18 months previously
General practitioner			≥2 times a week	
Psychologist			N/A	N/A

*HCP* healthcare provider, *MDD* major depressive disorder, *N/A* not applicable

#### Recruitment in Germany

All countries recruited equally for the IMPACT study with the exception of Germany, where recruitment was affected by issues related to study timelines and local review board approval. In Germany, only six depressed patients were recruited, three of whom were accompanied by a secondary participant (partner or friend) for part of the interview. In addition, no workplaces, colleagues, or HCPs were recruited in Germany.

### Data collection

The IMPACT study used a patient-centric research model to capture the experiences of patients with cognitive symptoms in MDD in everyday life. The implemented ethnographic methodologies included semi-structured interviews, observations of relevant activities, mapping exercises, and situation card exercises. Research was conducted in the home or office of the participant, depending on the participant type.

Interviews were conducted by trained ethnographers, with university degrees in the social and human sciences, from ReD Associates and consisted of a daylong visit to the patient’s home, with the secondary participant present for ≥1 h. HCP interviews lasted ~2 h and were conducted at their place of work. Coworker interviews lasted ≥1.5 h and were conducted at their place of work where possible. Interviews explored a range of themes dependent on the participant type: patients (Additional file 1: Table S1a), HCPs (Additional file 2: Table S1b), or colleagues (Additional file 3: Table S1c). Mapping exercises involved the patient drawing the evolution and components of their depression and the development of their cognitive symptoms; situation card exercises focused on determining the situations in which cognitive symptoms are most obvious by having the patient describe their cognitive complaints before and after the onset of their depression in the context of the situation presented on the card.

All interviews were recorded with the permission of the participant and field notes were written following each interview. All participant names and identifying information have been changed to protect privacy.

### Data analysis

We utilized the “sense making” method [24], which begins with the study of a phenomenon—in this case the experience of cognitive symptoms in depression. The data driving the study of the phenomenon are not quantitative in nature, but consist of pictures, emotions, artifacts, observed behavior, and conversations. A model of phenomenological explication inspired by Heidegger’s concept of a “formal indication” is then used to make sense and discover patterns in the data that have been gathered [25].

Our analysis began by a series of open, data-driven story-telling sessions. During these sessions, each researcher gave a thorough presentation of each of their respondents to the full project team, offering “thick descriptions” of their respondents’ behavior (i.e., detailed and specific), which illustrated the nuances of their culturally complex gestures [26]. These presentations drew on each researcher’s field notes, as well as audio and visual material from the interview. At the conclusion of the story-telling sessions, the researchers stepped away and independently made decisions about the key themes represented. The team then compared responses and collectively agreed on thematic categories into which the data would be coded, ensuring that the analysis was firmly grounded in data.

The researchers systematically coded all interviews according to these key thematic areas. When the coding was concluded, the researchers rigorously tested the thematic content of the codes by exploring similarities and differences between participants, and across research markets. Special attention was paid to examining unexpected findings, and active efforts were made to disprove prior interpretations. The findings are thus a result of an iterative process, in which the team routinely challenged its own assumptions and refined the analysis until consensus was achieved.

**Fig. 1:**
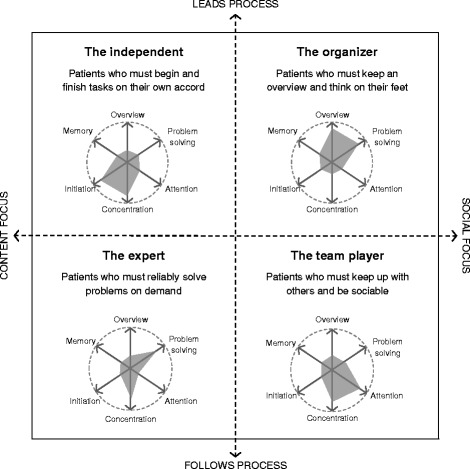
Work profiles

Using the ethnographic interviews, patient observation, mapping exercises, and situation card exercises, analysts derived four work profiles representing a variety of challenges for working professionals with cognitive symptoms in MDD. We spoke with each patient at length about their job, rating its cognitive requirements in nine key areas (concentrating/staying focused, processing information, memory, overview, verbal abilities, problem solving/decision making, attention control, motor skills and perception, and ability to initiate actions). After experimenting with different groupings to find meaningful correlations between job type (e.g. “people-centered” versus “knowledge-centered”), challenge areas (i.e. the largest discrepancies between the job’s cognitive requirements and the depressed patient’s cognitive performance), and consequences (i.e. what happened, both internally and externally, for the patient and others as a result of the challenge areas), clear patterns emerged by grouping along two axes: (1) whether the focus of the work is social or is based on content, and (2) whether the work involves leading or following processes (Fig. 1). Social focus jobs are those with significant interaction with others (e.g. account director, public relations specialist), whereas content focus jobs would involve less interaction and more focus on the project or deliverable at hand (e.g. architect, programmer). Leading versus following processes refers to whether an individual drives a team/project (e.g. medical director, police commissioner) or someone else in that team/project provides them direction (e.g. medical writer, police officer).

## Results

### Participant characteristics

In all, 34 depressed patients; 31 partners, relatives, or friends; 21 colleagues from 18 workplaces; and 13 independent HCPs participated in the study (Table 4). More than half of the participating MDD patients were women (19/34; 56%) and most were employed (30/34; 88%). The mean (±standard deviation) age of the depressed patients was 40 (±9.1) years. Demographic characteristics were similar across countries. Of the partners/relatives/friends interviewed, there were 13 spouses/partners, 5 parents, 2 children, 2 siblings, and 11 other close acquaintances (i.e. friend, neighbor, roommate, or extended family). Workplace colleagues included 14 coworkers, 3 superiors, 2 HR managers, and 2 HR representatives. The independent HCPs interviewed included 5 psychiatrists, 4 psychologists, and 4 general health practitioners.

**Table 4:** Study population of the IMPACT study by patient type and country (city)

Population interviewed, *n*	Brazil (Sao Paolo)	Canada (Toronto)	China (Shanghai)	France (Paris)	Germany (Berlin, Hamburg)	Total
Patients with MDD	7	7	7	7	6	34
Partners/Relatives/ Friends	7	7	7	7	3	31
Colleagues (Workplaces)	4 (4)	5 (4)	6 (4)	6 (6)	0	21 (18)
HCP	3	3	4	3	0	13

*HCP* healthcare provider; *MDD* major depressive disorder

### Patient perception of cognitive symptoms and the impact on daily life

#### The missing link between cognitive symptoms and depression

Most depressed patients had experienced cognitive symptoms before realizing that these might be related to their depression. Before discovering this relationship, many had attributed their symptoms to innate negative aspects of their personality, stress in the work environment, or family problems. Some even feared the presence of other disorders, such as Alzheimer’s disease. When Clara (age 23, France) experienced mental fogginess and vertigo, she *“didn’t realize that depression could have a physical and intellectual impact at the same time.”* When another patient, Maya (age 38, Canada), was first diagnosed with depression*, “people began to ask [her] if [she] had ADHD.”* She assumed that her cognitive symptoms *“might have to do with age,”* but this perception was corrected by her HCP.

Some patients remembered that discovering the relationship between their cognitive symptoms and their depression was a point of relief. As one patient stated:

*“It was a turning point for me when I found out that the problems* [cognitive symptoms] *were due to my depression. … [I was terrified of having something] much more serious—I thought I was going to die.” (Sara, age 49, Brazil)*

#### Impact on activities of daily living and close relatives

Patients in all five countries indicated their cognitive symptoms of depression (e.g. leaving the water running, forgetting the keys in the door, losing orientation, mixing up words) interfered with their activities of daily living (ADL). In addition to making mistakes and losing track of their activities during ADL, patients experienced mental exhaustion from activities they had formerly considered routine and were often unable to turn intentions into actions.

For many patients, difficulties with prioritizing tasks and keeping organized had a large impact on ADL, causing the tidiness of their homes to deteriorate*.* At one point, a single mother with two children found it too mentally overwhelming to shop for groceries and cook food for her children, so the family stopped eating together and everyone began to feed themselves. This depressed patient reported feeling like a bad mother and worried about how she was “hurting” her children by not being able to prepare meals or help them with homework and allowing them to form bad habits. Another patient’s inability to initiate and sustain action led him to do less work around the house, including planned home repairs and renovations. He used to enjoy and excel at home improvement, but after his cognitive symptoms emerged he had difficulty prioritizing and did not get around to doing anything unless he was explicitly asked. He and his girlfriend saw their home deteriorate around them and knew it to be a financial loss:

*“I’ll just sit there and do nothing. … We bought the house to fix it up. I looked forward to it. I haven’t done anything in more than a year. The windows need renovation, I know, but… it’s worth less now than when we bought it.” (Steve, age 51, Canada)*

The partners and relatives of patients with MDD in all five countries reported significant effects on interpersonal relationships. These individuals indicated they often had to take on additional tasks formerly performed by the patients and to contend with unpredictable emotions and behavior. In one case, the long-term girlfriend of a depressed patient experiencing cognitive symptoms related to initiation and concentration had taken over much of the housework that her boyfriend used to complete. She began feeling that her boyfriend had become someone she had to care for, like a child. He noticed how the relationship had changed and felt guilty about being a bad partner and a burden to his girlfriend.

#### Impact on social life and recreation

Many patients talked about the negative social and recreational consequences of having strained work life due to cognitive symptoms. One patient described how, exhausted by the effort it took to function with her cognitive symptoms at work, she had been isolating herself from social and leisure activities. To her, and to many other patients, going out with friends was not as important as work. In other cases, recreational activities were directly hindered by cognitive symptoms. For example, one patient explained that his cognitive symptoms hampered his ability to engage in his favorite hobby, playing multiplayer online games with his university friends. He now found gaming more difficult and could no longer anticipate other gamers’ moves when playing. This made playing less enjoyable and he had therefore withdrawn from this otherwise enjoyable and social hobby. A friend of this patient reported feeling that he had seemingly lost interest in the game and in them. He did not understand why his friend had become such a bad team member and felt rejected:

*“He used to be a really solid player. I don’t know what’s up with him. If he’s tired of playing, he should just quit. It’d be a shame to lose him; he’s a good friend, but it’d be better than it is now.” (China)*

#### Impact on professional life

In working patients, cognitive symptoms often interfered with their work performance. More than half of the patients interviewed (18/34; 53%) indicated they had changed jobs due to cognitive symptoms. Four other patients (12%) indicated they had considered changing jobs because of cognitive symptoms.

When describing their work performance, patients from all countries used words relating to forgetfulness, distractibility, and reduced problem solving—terms that are key indicators of cognitive symptoms in MDD. As one patient stated: *“At one point, I overlooked a meeting with a client. I simply stood him up. I can’t have something like that happen again.” (Ana, age 36, Brazil).*

According to another patient: *“If I’m in the middle of something and someone walks up to me and asks a question, then I have to start all over with the thing I was doing. I can’t just stop and then pick up where I left off. I have to start from the beginning; otherwise I’m lost.” (Beathe, age 43, Germany).*

Colleagues of depressed patients reported that the individuals with depression were less productive and made mistakes that damaged business outcomes. These effects created negativity and resentment, as well as fostered additional tensions among other workers in the same business unit. Allie, an administrator at a public health organization who used to have a depressed colleague, reports that other workers knew her colleague was depressed, “*but still found it really annoying that she didn’t do her job properly. … They thought that she was taking advantage of the situation.”*

### Four work profiles and the corresponding cognitive challenges

Cognitive symptoms in MDD are measured as a function of difficulties in processing speed, attention, memory, and executive functioning. In addition to the specific cognitive symptoms experienced by a patient, challenge areas and barriers in work life are determined by the demands of a patient’s job. Using the ethnographic interviews, patient observation, mapping exercises, and situation card exercises, analysts derived four work profiles (Organizer, Team Player, Expert, and Independent) representing the variety of challenges faced by depressed working professionals with cognitive symptoms. These work profiles represent the diversity of cognitive complaints in relation to work and highlight the interaction of cognitive symptoms on certain domains reflected in the profiles. The work profiles are theoretical constructs. Many workers will not fit into these distinctions in a clear-cut way and some might best be described by a combination of profiles. The patients described for each work profile are representative of not only the profile, but also demonstrate various responses to difficulties in key cognitive skills needed for work.

#### Organizer

The “Organizer” category included workers who had to keep an overview and needed to think on their feet (social focus and leads processes). Examples of Organizer positions would include managers, planners, project managers, and secretaries. These workers supervised many activities simultaneously and could quickly prioritize and solve issues as they emerged. When Organizers experienced cognitive symptoms (especially reductions in executive functioning, processing speed, and problem-solving skills), tasks piled up, leaving Organizers feeling confused and overwhelmed. Organizers often had few options for slowing down or catching up, and they could become anxious and exhausted as a result.

The Organizer profile was exemplified by a 36-year-old project manager from Brazil whose cognitive symptoms took the form of challenges with problem solving and gaining overview. At her multinational company, she began to experience difficulty with prioritizing tasks and keeping herself organized:

*“I’m such a mess now … I can’t keep an overview of anything. I can never find what I need. My mind is everywhere … I keep losing track of everything … I feel like I have to double-check everything. I can’t trust my intuition…”*

To compensate, she tried to speed up her work while simultaneously watching for errors, but this behavior was exhausting for her and she felt constantly on edge. She kept making mistakes and was unable to keep organized so her colleagues took on extra work in order to make up for her lost productivity. Although her colleagues appreciated her as a person and respected her experience and skills, they had grown weary of the extra work and became increasingly impatient with her as she began to lash out at them. Her boss eventually chose to let her go due to “problems collaborating.” Although it was likely a distorted interpretation of the situation, she thought of her many years of professional success as “pure luck” and that the shortcomings she experienced were intrinsic to her personality. She felt useless and was convinced that she would never be able to find another position at the same level; she was contemplating getting a job as a housekeeper.

#### Team player

The “Team Player” category of workers included those who had to keep up with others and be sociable (social focus and follows processes). Examples of Team Player positions would include social workers, police officers, and sales people. Team Players frequently interacted with other people, requiring them to filter new information and react quickly while following set procedures. When Team Players could no longer process their surroundings and focus on tasks (loss of concentration and attention skills), they became frustrated or irritable and could grow resentful of colleagues.

The Team Player profile was portrayed by a full-time nurse at an elder-care facility in Germany. At 43, she was a single mother of two children and had experienced recurrent MDEs throughout her life. She had begun experiencing attention and concentration problems, which caused her to be less productive at work. She became easily distracted, forgot information, made mistakes, and left projects half-finished. She also found it extremely difficult to resume tasks once interrupted. Because of these symptoms, she no longer felt that she could perform at the same level as her colleagues*.* Her colleagues tried to help by re-explaining and reminding her about tasks, but she often became a bottleneck and it was difficult for them to rely on her work. To adjust, her colleagues designed work processes so that they were not dependent on her contribution: *“I know that she has been suffering from depression and that she is doing what she can. It is just difficult for us, because we’re so busy already. … It’s less of a hassle to just do it yourself.”*

In an attempt to compensate for feeling that she was letting down her colleagues, she insisted that she take on extra night shifts; however, this extra commitment exacerbated her work problems. Frequently, she felt so exhausted when she woke up that she could not go to work and would call in sick at the last minute, further disappointing her colleagues.

#### Expert

The “Expert” work category included workers who had to reliably solve problems on demand (content focus and follows processes). Examples of Expert positions would include architects, tax consultants, and accountants. Experts parsed complex problems into smaller steps and communicated their knowledge to others in an accessible manner. Concentration and problem-solving deficits would make the Expert inefficient and sometimes incapable of performing their job, which would leave them feeling useless and uncertain of their skills. They might also worry about getting fired.

The Expert profile was illustrated by a 29-year-old engineer in a manufacturing plant in China. During his first MDE, he was experiencing cognitive symptoms, specifically with problem solving and concentration. As a highly specialized engineer, he had prided himself on his ability to solve problems that no one else at the factory was qualified to address, but he began struggling to deliver. He would begin working on a task but would get stuck, felt unsure of what algorithm to apply and when, spent time repeating tasks without finishing them, and began to feel like he “always gets the data wrong.” His concentration issues came to a head when he ordered incorrectly sized machine parts, resulting in a batch of parts worth thousands of dollars having to be discarded. As he had been a good employee for a long period of time, his boss sent him on sick leave rather than firing him. The head of his department described him as “in another world” and did not understand the engineer’s symptoms. His department head even felt relieved when he left on sick leave. Although he wanted to return to work and prove his worthiness, he feared another mistake would cost him his job. He wanted to be certain his abilities were back to normal before returning, but had lost trust in himself and did not know how to assess his mental improvement. As a result, he continued to stay at home: *“I thought I’d get fired. I’ve taken 3 months off. I can’t afford to make any more mistakes when I go back—I’m sure I’d be fired. I get so anxious thinking about it…”.*

#### Independent

The “Independent” category included workers who had to begin and finish tasks on their own accord (content focus and leads processes). Examples of Independent positions would include self-employed workers, freelancers, and students. In this type of work, patients had to develop and maintain their own structure for setting and reaching goals. Without structure, Independent workers procrastinated and avoided working. As they would struggle to initiate tasks, performance anxiety and fear of failure made it difficult to concentrate, which in turn could lead to uncompleted projects and low self-esteem.

The Independent profile was demonstrated by the owner of a graphic design company in Canada who had several MDEs—the most recent of which was 4 months prior to the interview. At the time of the interview, he was experiencing initiation and concentration difficulties that directly impacted his and his company’s performance. Before his cognitive symptoms emerged, he would dive right into projects, but now it took him several attempts to begin. He knew he should bid on more jobs but just could not seem to get the task done. He would sit down to write a proposal and end up procrastinating. As his once-booming business dwindled, his income went down as well, which threatened his and his girlfriend’s financial security. There was a real risk that they would have to sell their home, and his girlfriend felt pressure to earn more. Even though he was aware of his diminishing business, he did not proactively solve the problem and was unable to understand why he did not act. He felt guilty about neglecting his business and about the situation he was putting his girlfriend in, but his worries and deteriorating mood just made it more difficult to initiate and concentrate on tasks:

*“It’s not that I don’t have the will to work—I simply don’t have the wherewithal. I feel slow, and I start to neglect the everyday stuff. And then my self-esteem starts to fall. I never planned this when I started on my own. It’s certainly not what I dreamt of. It’s a terrible situation, and the guilt is just making everything worse.”*

### Healthcare provider insights

Responses from the 34 patients as well as the 13 independent HCPs interviewed for this study indicated that the practices for identifying cognitive symptoms in patients with MDD vary from country to country. For diagnosing depression, HCPs in Canada and China often used objective rating scales, such as the Hamilton Depression Rating Scale, the Personality Assessment Inventory, and the Beck Depression Inventory—or country-specific variants of these tests—whereas HCPs in Brazil and France tended to rely more on subjective evaluations in conversations with the patient. The depression assessments used by HCPs may contain specific items relating to cognitive symptoms, but they fail to measure the depth and impact of those symptoms. Cognitive symptoms were less commonly assessed via testing because scales specific to this aspect of MDD were perceived to be lacking or be too expensive. Additionally, the lack of treatments effective in treating cognitive symptoms in MDD was cited by some HCPs as a reason to forgo more specific assessment.

Overall, pharmacotherapy was perceived as the mainstay of MDD treatment; however, non-drug approaches—such as psychotherapy, traditional Chinese medicine, relaxation classes, and brain exercises (e.g. puzzles, card games, or video games)—were commonly prescribed to complement drug therapy in order for patients to return to normal functional levels in ADL. The use of supplementary medications with the specific goal of treating cognitive symptoms was rarely described (discussed by only one psychiatrist, in Canada). Nevertheless, regardless of country, HCPs cited cognitive symptoms in depression as a barrier to both therapy and engagement in everyday activities, particularly those required in the workplace. As such, HCPs viewed restoration of normal cognitive functioning as necessary for the treatment of depression, for patients to fully participate in therapy, and for patients to satisfactorily perform workplace functions requiring information processing and good judgment.

## Discussion

### Impact on home and work life

This ethnographic study provides insight into the common cognitive complaints of people with depression in five countries across the world and the negative impact of those symptoms on daily life, work, and social relations. In private life, cognitive symptoms seemed to cause relationship deterioration as the patients’ ability to contribute to the household diminished. In the workplace, consequences for others arose as patients made mistakes that hurt their company business and income and as colleagues were forced to take on additional work to compensate for patients’ cognitive symptoms. Work was a key source of identity, structure, and meaning, and patients tended to prioritize work over other spheres of life, with many continuing to work during depression. Cognitive symptoms often interfered with depressed patients’ work performance, which, in conjunction with the high priority patients tended to give to work, would imply that work life is very likely to contain the biggest challenge areas.

Significant relationships between cognitive functioning and home and workplace performance in MDD patients have been described previously. Saragoussi and colleagues reported the impact of patient-reported cognitive symptoms at baseline on quality of life, work, and overall functioning on a preliminary 1000-patient dataset from the PERFORM study [27]. In addition to the impact of depression severity on these outcomes, patient-reported cognitive complaints in depressed patients were associated with poorer quality of life, work productivity, and overall functioning. In their analysis of the relationships between various limitations and role functioning at home and at paid employment, Buist-Bouwman et al. concluded that approximately half of the impact of an MDE on role functioning was attributable to cognitive symptoms and feelings of embarrassment [28]. McIntyre et al. demonstrated a direct relationship between a subjective measure of cognitive symptoms and workplace performance variability that was greater than the relationship between depression symptom severity and workplace performance (standardized beta coefficients, 0.58 [*p* < 0.001] and 0.18 [*p* = 0.001], respectively) in their post hoc analysis of data from patients with MDD who participated in the International Mood Disorders Collaborative Project [12]. The results of the latter analysis suggest that cognitive symptoms in MDD may be a greater predictor of workplace performance variability than depressive symptom severity.

### The destructive cycle of depression and cognitive symptoms

**Fig. 2:**
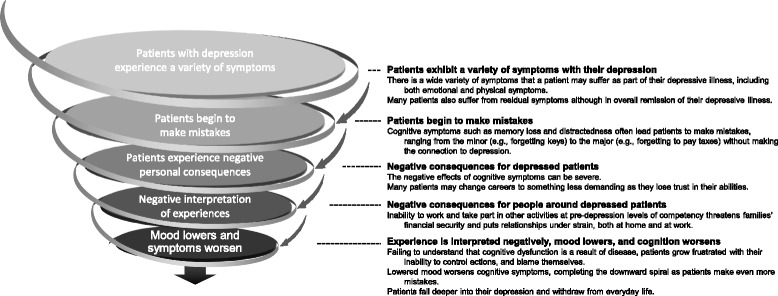
Modeling the impact of cognitive symptoms on the progression of major depressive disorder

A major finding of the IMPACT study is the interplay between depressive (mood) symptoms and cognitive symptoms (Fig. 2). Patients did not typically associate their cognitive symptoms with their depression, although these deficits can lead to a loss of functional ability that damages patients’ lives and can have negative consequences for people close to the patient. The negative experience of cognitive symptoms for the patient (e.g. feeling like a burden to others, losing one’s job) increased their stress and lowered their mood, which then created more stress and worsened cognitive symptoms, thereby creating a destructive cycle. This escalation can lead to further disengagement from the world. The patients exemplifying the derived work profiles demonstrated that this destructive cycle can exist within a variety of lifestyles, work types, and situations.

Currently, the medical focus is on reducing cognitive symptoms as “noise” or as an associated symptom of depression, rather than breaking the destructive cycle that occurs in the interaction between mood and cognitive functioning. Based on our observations, we recommend that HCPs focus more specifically on cognitive symptoms and how they impact patients’ lives and perception of themselves. The goal should be to raise patients’ awareness of the role of depression-related cognitive symptoms and help them overcome or, to some degree, compensate for these symptoms.

### Implications for treatment

Although HCPs viewed cognitive symptoms in MDD as a barrier for patients re-establishing their lives, the results of our survey suggest that assessment and treatment of cognitive symptoms is not common practice in patients with MDD. Belgaied and colleagues reported similar findings in their survey of psychiatrists from six countries (*N* = 61) in which 38% of respondents indicated that they did not routinely use information collected in the patient history interview to determine cognitive functioning in patients with MDD [29]. In addition, psychiatric re-evaluation of cognitive functioning occurred an average of 19 weeks (~5 months) during acute phase episodes and 45 weeks (~11 months) in the chronic phase [29].

**Fig. 3:**
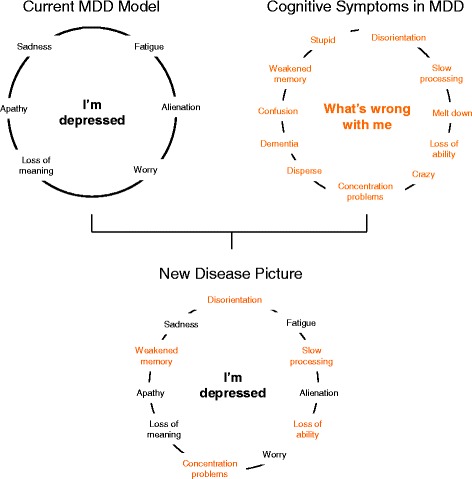
Introducing cognitive symptoms into the depression model

Currently, there is no uniform vocabulary for the articulation of cognitive symptoms in MDD, though the results of this study suggest that cognitive symptoms of MDD are experienced similarly across a range of countries differing in language, ethnicity, and culture (Additional file 4). However, patients do not usually independently associate their cognitive symptoms with their depression, leading to an ultimate breakdown of communication: patients do not report their cognitive symptoms, and HCPs must rely on patient history to determine a patient’s cognitive status. Introducing the commonly used terms associated with cognitive complaints into the mental model of MDD will not only give patients, their partners/relatives/friends, and their colleagues a better understanding of MDD as a disease, but will also give them hope to know that their symptoms are real and are treatable (Fig. 3).

For the patients in the study, work was a key source of identity, structure, and meaning. Many patients continued to work during MDEs and were able to describe their cognitive symptoms most specifically in the context of the workplace. Patients found it difficult to answer abstract and/or direct questions about their cognitive symptoms, so it may be more prudent to instead ask about the negative consequences of such symptoms in everyday life, including work. Focusing on challenge areas that likely result from cognitive symptoms in a specific context can make it easier for patients to discuss cognitive symptoms and, through this, easier for HCPs to uncover these symptoms. If HCPs understand how cognitive symptoms manifest themselves in work life and at home, they will be better equipped to ask the right questions to prompt patients to bring up their cognitive symptoms in the consultation. This in turn may improve both diagnosis and treatment outcomes. Asking specific, narrowly tailored questions about workplace performance may complement formal diagnosis, capturing the nuance of patients’ illness. Differentiation would allow HCPs to diversify treatment and target the cognitive complaints of most importance to each patient case. In this way, the study shows the challenges and barriers of working with cognitive symptoms but also provides guidance for recovery strategies. Specifically, the findings indicate that new treatments that directly target cognitive symptoms are needed for patients with MDD.

Notably, the negative impact of cognitive symptoms on ADL in patients with MDD was present even during prescription MDD therapy, as all patients in this study had to self-report and verify a current prescription medication. But various antidepressants have been shown to enhance cognitive functioning in clinical trials, demonstrating beneficial effects on attention, learning and memory, processing speed, and/or executive functioning [30, 31]. Still, in several other clinical trials, an observed beneficial effect of antidepressant treatment on cognitive functioning did not correlate with improvements in depressive symptoms, captured with the Montgomery–Åsberg Depression Rating Scale and Hamilton Depression Rating Scale [32–37]. Furthermore, newer classes of antidepressants may be less likely to cause sedation-related functional impairment, which is sometimes associated with older classes of antidepressant medications [38–40]. A better understanding of these disparate effects could improve the overall management of patients with MDD by facilitating the treatment of cognitive symptoms in addition to depressive symptoms, thereby potentially improving functional outcomes.

### Limitations

The data collected in the IMPACT study were designed to illustrate the real-life experiences of patients with cognitive symptoms associated with MDD. These findings must, however, be interpreted in light of the limitations and potential for bias in the study design. First, no standard recruitment method was employed to identify study participants; rather, patients were recruited through a variety of methods in the different countries, which introduces selection bias. Various levels of depression and cognitive symptoms were likely represented, as there was no independent formal diagnosis made at the time of the study; patients self-reported having been diagnosed with depression, having cognitive symptoms, and being on an antidepressant. Both the colleague and the HCP participants were recruited separately from the depressed patients; therefore, their opinions, while applicable to the general depressed population, were not specific to the patients included in the study. Furthermore, colleagues of depressed patients were interviewed in the work environment, which may have influenced their responses. Interviews with HCPs and partners/relatives/friends were conducted in only four of the five countries. All interviews were conducted in the local language and then translated; thus the results were open to interpretation. Notably, the study involved only *subjective* reports of cognitive symptoms. Given the poor correlation between subjective and objective measures of cognition, with subjective problems being largely influenced by depressive symptoms [41, 42], this limits the conclusions of the study. However, the study was qualitative in nature, aiming to examine how patients’ *experienced* cognitive symptoms impacted on domains in their lives and people around them. This qualitative research was of interest to the sponsoring corporation because, at the time of this study, the sponsor was evaluating the impact of their antidepressant product (vortioxetine) on patients with MDD and cognitive symptoms. Lastly, the economic impact of cognitive symptoms in these depressed patients is not known, as no cost correlation was conducted.

## Conclusions

In summary, this study provides additional insight into the presence of cognitive symptoms in patients with MDD and its (experienced) negative consequences on daily life, personal relationships, and workplace productivity and on worsening depression. The study shows that the lack of awareness of cognitive symptoms as part of depression lays the groundwork for how cognitive symptoms in MDD interact with mood. Acknowledging and targeting the cognitive symptoms in MDD could potentially help patients break the destructive cycle that pulls them deeper into depression and help them return to everyday life.

## Additional files

Additional file 1: Table S1a. Research themes and questions for patients, family, and close friends. (DOCX 51 kb)

Additional file 2: Table S1b. Research themes and questions for healthcare providers. (DOCX 51 kb)

Additional file 3: Table S1c. Research themes for colleagues of depressed patients. (DOCX 48 kb)

Additional file 4: Terminology surrounding cognitive difficulties. (DOCX 49 kb)

## Abbreviations

HCP: Healthcare provider
HR: Human resources
ICC/ESOMAR: International Chamber of Commerce/European Society for Opinion and Marketing Research
IMPACT: Investigating MDD Patients’ Accounts of Cognitive Symptoms During Treatment
MDD: Major depressive disorder
MDE: Major depressive episode
MHP: Mental health professional
N/A: Not applicable
