# Supplementary material for: An ethnographic study of the effects of cognitive symptoms in patients with major depressive disorder: the IMPACT study
Source: BMC Psychiatry. 2017 Nov 21;17:370. doi: 10.1186/s12888-017-1523-8 (PMC5697414; doi:10.1186/s12888-017-1523-8)
Supplement: Supplementary file 1 — Research themes and questions for patients, family, and close friends. (DOCX 51 kb) [file 12888_2017_1523_MOESM1_ESM.docx]

**Table S1a** Research themes and questions for patients, family, and close friends

| **Focus** | **Theme** | **Key research questions** |
| --- | --- | --- |
| Experience of cognitive dysfunction  in each aspect  of life | Work life | - How does cognitive dysfunction become visible in the workplace? - How do patients and those they work with handle cognitive dysfunction? - What are the hopes, fears, and challenges around absence and return to work? |
|  | Family life | - How does cognitive dysfunction manifest itself in everyday life with close family? - How is cognitive dysfunction talked about or otherwise approached by family members – if at all acknowledged? |
|  | Leisure and social life | - How does cognitive dysfunction influence your ability to connect to friends? - What is the impact of cognitive dysfunction on leisure activities? - What are the struggles around leisure and social life during sickness absence and return to work? |
|  | Inner life | - What is the lived experience of being confused, forgetful, and indecisive? Is it perceived as integral to the MDD? - How does cognitive dysfunction influence your sense of who you are? - How does cognitive dysfunction affect aspirations and purpose? |
|  | Medical history | - What is patient’s history with depression? - What is patient’s history with cognitive dysfunction? - What is patient’s treatment history: medical, pharmacological, and other? - What is the relation between treatment and cognitive dysfunction? |
| MDD, major depressive disorder. | | |
