# Supplementary material for: An ethnographic study of the effects of cognitive symptoms in patients with major depressive disorder: the IMPACT study
Source: BMC Psychiatry. 2017 Nov 21;17:370. doi: 10.1186/s12888-017-1523-8 (PMC5697414; doi:10.1186/s12888-017-1523-8)
Supplement: Supplementary file 2 — Research themes and questions for healthcare providers. (DOCX 51 kb) [file 12888_2017_1523_MOESM2_ESM.docx]

**Table S1b** Research themes and questions for healthcare providers

| **Focus** | **Theme** | **Key research questions** |
| --- | --- | --- |
| Perception of cognitive dysfunction and its relationship to MDD | Belief system around MDD and cognitive dysfunction | - How do MHPs think about the relationship between mood and cognition? |
|  | Perception of impact of cognitive dysfunction on patients’ lives | - What do MHPs see as the biggest problems for those of their patients who experience cognitive dysfunction? |
|  | Attitude towards sickness absence and return to work | - What is the outlook of MHPs on sickness absence and return to work, and how much emphasis do they place on cognitive dysfunction in thinking about this? |
| Clinical practices around cognitive dysfunction and MDD | Identifying and evaluating cognitive dysfunction | - How do MHPs identify cognitive dysfunction among their MDD patients? - How, if at all, do they attempt to measure cognitive functioning? |
|  | Discussing and advising on cognitive dysfunction with patients | - What are the issues around talking to patients about their cognitive functioning? - What vocabulary is used to discuss cognition in the clinic? |
|  | Role of cognitive dysfunction in MDD treatment approach | - How does cognitive dysfunction factor into overall decision-making about treatment? - What cognitive impact do MHPs believe existing drugs to have? - What, if any, specific treatment options are recommended for cognitive dysfunction? |
| MDD, major depressive disorder; MHP, mental health professional. | | |
