# Supplementary material for: An ethnographic study of the effects of cognitive symptoms in patients with major depressive disorder: the IMPACT study
Source: BMC Psychiatry. 2017 Nov 21;17:370. doi: 10.1186/s12888-017-1523-8 (PMC5697414; doi:10.1186/s12888-017-1523-8)
Supplement: Supplementary file 3 — Research themes for colleagues of depressed patients. (DOCX 48 kb) [file 12888_2017_1523_MOESM3_ESM.docx]

**Table S1c** Research themes for colleagues of depressed patients

| **Focus** | **Theme** | **Key research questions** |
| --- | --- | --- |
| Experience of cognitive dysfunction in each aspect of life | Appearance in the workplace | - How does depression show in the workplace? - How does cognitive dysfunction show in the workplace? - Who (job types, positions) notices what? |
|  | Reactions & strategy | - How do colleagues and superiors react to depression and cognitive dysfunction? - What strategies do colleagues, superiors, and HR departments employ to deal with depression and cognitive dysfunction? - What assumptions about depression and cognitive dysfunction drive perception in the workplace? |
|  | Cognitive dysfunction and work types | - How does depression and cognitive dysfunction affect different types of work? - What cognitive symptoms (e.g. ability to concentrate, forgetfulness) in particular affect different types of work? |
|  | Mapping consequences | - What are the implications on patients’ own productivity? - What are implications and tasks for colleagues, superiors, and HR of working with a person with cognitive dysfunction? - What are the calculations and economic logistics that guide strategy and reaction to cognitive dysfunction in the workplace? |
| HR, human resources. | | |
