# Supplementary material for: An ethnographic study of the effects of cognitive symptoms in patients with major depressive disorder: the IMPACT study
Source: BMC Psychiatry. 2017 Nov 21;17:370. doi: 10.1186/s12888-017-1523-8 (PMC5697414; doi:10.1186/s12888-017-1523-8)
Supplement: Supplementary file 4 — Terminology surrounding cognitive difficulties. (DOCX 49 kb) [file 12888_2017_1523_MOESM4_ESM.docx]

*Terminology surrounding cognitive difficulties*

Patients with MDD from the different continents showed slightly different ways of talking about their cognitive difficulties; however, they tended to describe their symptoms using everyday terms when asked to articulate their experience. In Canada, phrases such as *“scattered,” “meltdown,” “falling apart,”* and *“lost”* were used to describe cognitive impairment. Participants in France used phrases like *“mes oublis” (my oversights), “épuisement professional” (burn out), “grande fatigue” (great tiredness)*, and *“fou” (crazy)*. In Germany, patients commonly referred to experiencing cognitive difficulties as *“dämlich” (stupid), “chaotisch” (chaotic), “kontrollverlust” (loss of control)*, and *“schusseligkeit” (scattiness)*. Brazilian patients used phrases such as *“disperso” (disperse), “confusão” (confusion), “eu viajo” (my thoughts wander), “eu penso à tôa” (I think randomly), “eu fico distraído” (I get distracted),* and *“sem iniciativa” (without initiative)*, and in China, words included*记忆力减退 (memory loss), 无法集中注意力 (cannot concentrate), 自己没用 (useless), 变笨 (become stupid),* and *反应慢 (slow reaction)*.

Although the vocabulary differed in each country, most patients expressed symptoms that seemed to reflect one or more of the four cognitive domains that are often compromised by MDD: processing speed, attention, memory, and executive functioning (Papakostas, 2014).

**Reference**

[1] Papakostas GI. Cognitive symptoms in patients with major depressive disorder and their implications for clinical practice. J Clin Psychiatry. 2014;75:8-14.
